# Multivessel versus culprit-only PCI in STEMI patients with multivessel disease: meta-analysis of randomized controlled trials

**DOI:** 10.1007/s00392-020-01637-6

**Published:** 2020-04-01

**Authors:** Hans-Josef Feistritzer, Alexander Jobs, Suzanne de Waha-Thiele, Ingo Eitel, Anne Freund, Mohamed Abdel-Wahab, Steffen Desch, Holger Thiele

**Affiliations:** 1 Department of Internal Medicine/Cardiology, Heart Center Leipzig at University of Leipzig, Strümpellstr. 39, 04289 Leipzig, Germany; 2 Leipzig Heart Institute, Leipzig, Germany; 3 Department of Cardiology, Angiology and Intensive Care Medicine, University Heart Center Lübeck, University Hospital Schleswig-Holstein, Lübeck, Germany

**Keywords:** ST-elevation myocardial infarction, Multivessel coronary artery disease, Culprit vessel, Revascularization

## Abstract

**Aims:**

To perform a pairwise meta-analysis of randomized controlled trials (RCTs) comparing multivessel percutaneous coronary intervention (PCI) and culprit vessel-only PCI in ST-elevation myocardial infarction (STEMI) patients without cardiogenic shock.

**Methods:**

We searched MEDLINE, Cochrane Central Register of Controlled Trials, and Embase for RCTs comparing multivessel PCI with culprit vessel-only PCI in STEMI patients without cardiogenic shock and multivessel coronary artery disease. Only RCTs reporting mortality or myocardial reinfarction after at least 6 months following randomization were included. Hazard ratios (HRs) were pooled using random-effect models.

**Results:**

Nine RCTs were included in the final analysis. In total, 523 (8.3%) of 6314 patients suffered the combined primary endpoint of death or non-fatal reinfarction. This primary endpoint was significantly reduced with multivessel PCI compared to culprit vessel-only PCI (HR 0.63, 95% confidence interval [CI] 0.43–0.93; *p* = 0.03). This finding was driven by a reduction of non-fatal reinfarction (HR 0.64, 95% CI 0.52–0.79; *p* = 0.001), whereas no significant reduction of all-cause death (HR 0.77, 95% CI 0.44–1.35; *p* = 0.28) or cardiovascular death (HR 0.64, 95% CI 0.37–1.11; *p* = 0.09) was observed.

**Conclusions:**

In STEMI patients without cardiogenic shock multivessel PCI reduced the risk of death or non-fatal reinfarction compared to culprit vessel-only PCI.

**Electronic supplementary material:**

The online version of this article (10.1007/s00392-020-01637-6) contains supplementary material, which is available to authorized users.

## Introduction

Approximately 50% of patients with ST-elevation myocardial infarction (STEMI) present with multivessel coronary artery disease at the time of primary percutaneous coronary intervention (PCI), which is associated with worse prognosis [1–3]. The optimal management of additional stenoses in non-culprit coronary arteries is still under debate. While earlier observational studies reported worse outcomes with multivessel revascularization performed during primary PCI, numerous randomized controlled trials (RCTs) conducted during the last years suggested a benefit of multivessel PCI over culprit vessel-only PCI [4–10]. However, in several moderately-sized trials positive results were primarily driven by a reduced rate of subsequent myocardial revascularization rather than a reduction of hard clinical events [8, 9]. Recently, the so far largest COMPLETE (The Complete versus Culprit-Only Revascularization Strategies to Treat Multivessel Disease after Early PCI for STEMI) trial reported a significant reduction of the combined endpoint of cardiovascular death and new myocardial infarction [10].

We aimed to perform a meta-analysis of RCTs comparing the efficacy of multivessel revascularization compared to culprit vessel-only PCI in STEMI patients without cardiogenic shock.

## Methods

### Search strategy and selection criteria

We searched MEDLINE through PubMed (up to July 22, 2019), Cochrane Central Register of Controlled Trials (up to July 22, 2019), and Embase (up to July 25, 2019) for RCTs of potential interest. Only studies published in English language were considered. Four groups of search terms were used, of which at least one term in each group was required to match: (1) "STEMI", "ST-elevation myocardial infarction", "ST-segment elevation myocardial infarction", "ST-elevation myocardial infarction", "ST-segment elevation myocardial infarction", and “ST-elevation"; (2) “multivessel”, “multi vessel”, “multi-vessel”, “complete”, and “culprit”; (3) "PCI", “PPCI", "pPCI", "primary PCI", "percutaneous coronary intervention", "primary percutaneous coronary intervention", "revascularization", “revascularization", and "angioplasty"; (4) "randomized", "randomised", "random", and "randomly" (Online Supplement Section 1). The data of the COMPLETE trial were included in our analysis immediately after online publication [10].

We included RCTs comparing culprit vessel-only PCI versus multivessel PCI in patients with STEMI and multivessel coronary artery disease, reporting mortality or non-fatal myocardial infarction after at least 6 months following randomization. Multivessel revascularization could be performed immediately during the index procedure or staged, but within 2 months following culprit vessel PCI. RCTs including STEMI patients with cardiogenic shock as well as studies with staged procedures not within the above-mentioned timeframe were excluded. We excluded also RCTs which were not published in full text since the risk of bias and other critical features cannot be excluded. The present meta-analysis was registered at the PROSPERO international prospective register of systemic reviews (CRD42019142643).

### Data extraction and analysis

After removing duplicates, titles and abstracts of the identified studies were screened for eligibility by two independent observers (HJF and AF). In case of uncertainty, full-text articles were reviewed. Any discrepancies were resolved by discussion after consultation of a third investigator (AJ).

The risk of bias of the included trials was assessed by two independent investigators (HJF and AF) according to the Cochrane Collaboration’s tool for assessing risk of bias in randomized trials [11]. Again, after consultation of a third investigator (AJ), any discrepancies were resolved by discussion.

Data on sample size, length of follow-up, revascularization strategy, medical history, baseline clinical characteristics, and outcome were independently extracted by two observers (HJF and AJ). After checking for discrepancies and plausibility, individual study results were merged in a single uniformly coded data sheet.

### Outcomes

The primary endpoint was a combination of death and non-fatal myocardial reinfarction. Because of diverse definition of the combined endpoint (all-cause death vs. cardiovascular death) among the included trials, we performed additional analyses exclusively including studies reporting all-cause or cardiovascular death. Secondary endpoints included all-cause death, cardiovascular death, non-fatal myocardial reinfarction, and revascularization. In stratified analyses, we investigated the heterogeneity introduced by pooling studies investigating (a) different strategies for multivessel PCI (i.e. multivessel PCI within index procedure versus multivessel PCI as staged procedure), (b) fractional flow reserve (FFR)-guided versus angiography-guided multivessel PCI, and (c) all-cause death versus cardiovascular death as part of the composite endpoint of death or non-fatal myocardial reinfarction.

### Data analysis

We analyzed data by the intention-to-treat principle. Extracted hazard ratios (HRs) were converted to natural logarithms of HRs *lnHR.* The variance of lnHR V* was calculated using the extracted 95% confidence intervals of the HRs according to Eq. (1). In case HRs with respective 95% confidence intervals were not reported, we estimated these measures. For each treatment group *i* the failure rate *λ* was calculated using the number of events in that group* events*_*i*_, the number of patients in that group* patients*_*i*_, and the mean follow-up duration in months for the total population according to formula (2). The estimated HR resulted by dividing *λ* of the multi-vessel group by *λ* of the single vessel group as shown in formula (3). For the estimated HR V* was estimated according to formula (4):

1 $$V^* = \left[ {\frac{{\ln ({\text{upper}}\,95\% \,{\text{CI}}) - \ln ({\text{lower}}\,95\% \,{\text{CI}})}}{{2 \times 1.96}}} \right]^{2}$$

2 $$\lambda _{i}  = \frac{{\ln \left( {1 - \frac{{{\text{events}}_{i} }}{{{\text{patients}}_{i} }}} \right)}}{{{\text{duration~of~mean~follow-up}}}}$$

3 $${\text{HR}}_{{{\text{estimated}}}}  = \frac{{\lambda _{{{\text{multi-vessel}}\,{\text{group}}}} }}{{\lambda _{{{\text{culprit-vessel}}\,{\text{only}}\,{\text{group}}}} }}$$

4 $$V_{{{\text{estimated}}}}^{*}  = \frac{1}{{\sqrt {\frac{{{\text{events}}_{{{\text{multi-vessel}}\,{\text{group}}}}  \times {\text{events}}_{{{\text{culprit-vessel}}\,{\text{only}}\,{\text{group}}}}  \times {\text{events}}_{{{\text{total}}}} }}{{\left( {{\text{events}}_{{{\text{multi-vessel}}\,{\text{group}}}}  \times {\text{events}}_{{{\text{culprit-vessel}}\,{\text{only}}\,{\text{group}}}} } \right)^{2} }}} }}.$$

The* lnHR* published in the original articles showed good agreement with the estimated* lnHR* (Online Supplement Section 2). We, therefore, felt confident to use this approach to estimate the* lnHR* of studies not reporting HR in their original article. Study-level results (i.e.* lnHR* and V*) were pooled by means of a random-effects meta-analysis using the inverse variance method as primary analysis. Between-study variances *τ*^2^ was calculated according to Paule–Mandel with Hartung–Knapp adjustment. In addition, a fixed effect meta-analysis was calculated. Cochran’s *Q* statistic and Higgins and Thompsons *I*^2^ were calculated to assess heterogeneity. The presence of small-study effects was investigated visually by means of funnel plots. Since the number of included trials was below 10, we did not apply formal test of funnel plot asymmetry [12]. We evaluated interactions in stratified analyses in random-effects models combined from the final results.

## Results

### Trial characteristics

**Fig. 1:**
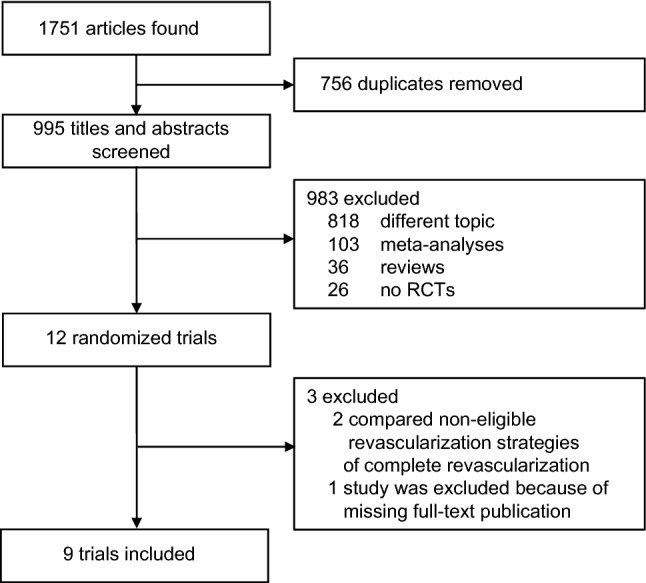
Trial selection. *RCT* randomized controlled trial

Our search resulted in 1751 search items including 756 duplicates. Detailed trial selection is demonstrated in Fig. 1. Screening of titles and abstracts identified 12 RCTs, of which 9 trials were included in final analysis (Fig. 1, Table 1). Two trials compared revascularization strategies not eligible for our meta-analysis and were, therefore, excluded (complete revascularization versus stress-echocardiography-guided revascularization in Calvino Santos et al. and staged-complete revascularization versus ischemia-driven revascularization in Mashhour et al.) [13, 14]. Another study (PRAGUE-13) was excluded because of missing full-text publication [15]. Definitions of study endpoints of the included trials are summarized in the Online Supplement Section 3.

**Table 1:** Key features of included trials

	Major inclusion criteria	Multivessel group	Culprit-only group	Primary endpoint
HELP AMI [27]	Multivessel CAD with the technical possibility of stent angioplasty in a major non-IRA	Immediate multivessel PCI of all suitable lesions	Culprit vessel-only PCI Subsequent interventions were performed at the investigator’s discretion	Repeat revascularization
Politi et al. [16]	Multivessel CAD, defined as > 70% diameter stenosis of two or more epicardial coronary arteries or their major branches by visual estimation	Two strategies: (1) Staged multivessel PCI (56.8 ± 12.9 days after primary PCI) (2) Multivessel PCI during the same procedure	Culprit vessel-only PCI	Combination of cardiac or non-cardiac death, in-hospital death, reinfarction, rehospitalization for acute coronary syndrome and repeat coronary revascularization
Ghani et al. [28]	Multivessel CAD, defined as one or more significant stenoses in at least two major epicardial coronary arteries, or the combination of a side branch and a main epicardial vessel provided that that they supplied different territories	FFR-guided (< 0.75) multivessel PCI during index hospitalization or within 3 weeks after STEMI. PCI was performed without preceding FFR measurement in severe lesions (> 90%)	Culprit vessel-only PCI Revascularization of non-IRAs in asymptomatic patients was discouraged	Combination of death, non-fatal reinfarction and additional revascularization
PRAMI [6]	Successful treatment of the IRA and ≥ 50% stenosis in one or more coronary arteries other than the IRA	Immediate multivessel PCI in non-IRAs with ≥ 50% stenosis	Culprit vessel-only PCI	Composite of cardiovascular death, non-fatal myocardial reinfarction or refractory angina
CvLPRIT [7]	Multivessel CAD: at least 1 lesion with > 70% in one plane or > 50% in 2 planes	Multivessel PCI including all non-IRAs during index admission. Single-stage complete revascularization was recommended	Culprit vessel-only PCI	Composite of all-cause death, reinfarction, heart failure and ischemia-driven revascularization
DANAMI-3-PRIMULTI [8]	> 50% angiographic diameter stenosis in one or more non-IRA	FFR-guided (≤ 0.80) multivessel PCI of all significant coronary lesions not related to the IRA 2 days after initial PCI	Culprit vessel-only PCI	Composite of all-cause death, reinfarction or ischemia-driven revascularization of lesions in non-IRAs
Hamza et al. [29]	Multivessel CAD with at least 80% stenosis in one or more non-IRA	Multivessel PCI of all non-IRAs during index procedure (recommended) or staged within 72 h of presentation	Culprit vessel-only PCI	Composite of all-cause death, reinfarction, and ischemia-driven revascularization by PCI or CABG
COMPARE-ACUTE [9]	≥ 50% diameter stenosis of one or more non-IRAs	FFR-guided (≤ 0.80) multivessel PCI, generally during index PCI, but could be performed staged before discharge	Culprit vessel-only PCI. FFR measurements of non-IRA lesions were performed but not used for decision making with respect to PCI	Composite of all-cause death, reinfarction, any revascularization and cerebrovascular events
COMPLETE [10]	≥ 70% stenosis by visual estimation or 50–69% by visual estimation and accompanied FFR ≤ 0.80	Staged multivessel PCI of all suitable non-culprit lesions irrespective of symptoms or evidence of ischemia Stratified by timing of non-culprit PCI: during index hospitalization vs. after discharge but no later than 45 days after randomization	Culprit vessel-only PCI, regardless of whether there was evidence of ischemia	Two co-primary endpoints: (1) composite of cardiovascular death or new myocardial infarction; and (2) composite of cardiovascular death, new myocardial infarction or ischemia-driven revascularization

*CABG* coronary artery bypass grafting, *CAD* coronary artery disease, *FFR* fractional flow reserve, *IRA* infarct-related artery, *PCI* percutaneous coronary intervention

Five out of 9 trials included showed a low risk of bias, whereas in 4 trials the risk of bias was substantial (Online Supplement Section 4). Additional quality indicators mirror the findings of the established risk assessment (Online Supplement Section 5). Key features of the included trials are summarized in Table 1. Baseline and treatment characteristics are shown in Table 2. With the exception of one study (Politi et al.), which compared culprit vessel-only revascularization with two different strategies of complete revascularization, patients were randomized to two different revascularization strategies in all trials (Table 1) [16]. The two groups receiving complete revascularization in the study by Politi et al. (immediate PCI of non-infarct-related arteries vs. staged PCI of non-infarct-related arteries) were combined for primary outcome analysis. The duration of follow-up ranged between 6 and 36 months among the included trials, whereas mean or median follow-up was only reported in 5 of 8 trials (Online Supplement Section 5). The other trials reported only the planned fixed follow-up duration ignoring censoring due to experiencing an endpoint event or loss to follow-up. Four trials did not report outcome data regarding the combination of death and non-fatal myocardial reinfarction and therefore could not be included in the analysis of the primary endpoint (HELP AMI, Politi et al. Ghani et al. and Hamza et al.). Six studies were included in the analyses of all-cause and cardiovascular mortality. The rate of myocardial reinfarction was reported in 8 trials.

**Table 2:** Baseline and treatment characteristics of included trials

	HELP AMI	Politi et al.	Ghani et al.	PRAMI	CvLPRIT	DANAMI-3-PRIMULTI	Hamza et al.	COMPARE-ACUTE	COMPLETE
Patients MV PCI (*n*)	52	130	80	234	150	314	50	295	2016
Patients CVO PCI (*n*)	17	84	41	231	146	313	50	590	2025
Age MV PCI (mean, SD or median, IQR; years)	63.5 ± 12.4	64 ± 11	62 ± 10	62^c^	65 ± 11	64 (37–94)	56.4 ± 11.5	62 ± 10	61.6 ± 10.7
Age CVO PCI (mean, SD or median, IQR; years)	65.3 ± 7.4	67 ± 13	61 ± 11	62^c^	65 ± 12	63 (34–92)	52.2 ± 10.6	61 ± 10	62.4 ± 10.7
Male MV PCI (*n*, %)	46 (88)	102 (78)	64 (80)	177 (76)	128 (85)	251 (80)	41 (82)	233 (79)	1623 (81)
Male CVO PCI (*n*, %)	14 (82)	64 (76)	33 (80)	186 (81)	112 (77)	255 (81)	43 (86)	450 (76)	1602 (79)
Diabetes MV PCI (*n*, %)	6 (12)	21 (16)	5 (6)	35 (15)	19 (13)	29 (9)	50 (100)	43 (15)	385 (19)
Diabetes CVO PCI (*n*, %)	7 (41)	20 (24)	2 (5)	48 (21)	20 (14)	42 (13)	50 (100)	94 (16)	402 (20)
Dyslipidemia MV PCI (*n*, %)	21 (40)	–	12 (15)	–	41 (27)	–	24 (48)	95 (32)	764 (38)
Dyslipidemia CVO PCI (*n*, %)	9 (53)	–	12 (29)	–	34 (23)	–	21 (42)	176 (30)	797 (39)
Hypertension MV PCI (*n*, %)	19 (37)	74 (57)	21 (26)	94 (40)	54 (36)	130 (41)	13 (26)	136 (46)	982 (49)
Hypertension CVO PCI (*n*, %)	10 (59)	50 (60)	17 (41)	93 (40)	51 (35)	146 (47)	18 (36)	282 (48)	1027 (51)
Prior MI MV PCI (*n*, %)	–	–	5 (6)	19 (8)	7 (5)	17 (5)	5 (10)	22 (7)	148 (7)
Prior MI CVO PCI (*n*, %)	–	–	2 (5)	16 (7)	5 (3)	27 (9)	3 (6)	48 (8)	154 (8)
Prior stroke MV PCI (*n*, %)	–	–	0 (0)	10 (4)	–	–	–	10 (3)	64 (3)
Prior stroke CVO PCI (*n*, %)	–	–	1 (2)	10 (4)	–	–	–	26 (4)	62 (3)
Contrast media MV PCI (mean, SD or median, IQR; ml)	341 ± 163	–	–	–	250 (190–330)	280 (215–365)	–	224 ± 104	–
Contrast media CVO PCI (mean, SD or median, IQR; ml)	242 ± 106	–	–	–	190 (150–250)	170 (125–220)	–	202 ± 75	–
Discharge medication									
Clopidogrel MV PCI (*n*, %)	–	126 (97)	–	–^d^	59^a^ (39)	43 (14)	–	78^a^ (26)	516 (26)
Clopidogrel CVO PCI (*n*, %)	–	71 (85)	–	––^d^	54^a^ (37)	38 (12)	–	154^a^ (26)	572 (28)
Ticagrelor MV PCI (*n*, %)	–	–	–	–^d^	19^a^ (13)	73 (23)	–	101^a^ (34)	1298 (64)
Ticagrelor CVO PCI (*n*, %)	–	–	–	–^d^	18^a^ (12)	67 (21)	–	209^a^ (35)	1281 (63)
Prasugrel MV PCI (*n*, %)	–	–	–	–^d^	58^a^ (39)	194 (62)	–	100^a^ (34)	193 (10)
Prasugrel CVO PCI (*n*, %)	–	–	–	–^d^	64^a^ (44)	204 (65)	–	197^a^ (33)	169 88)
DES MV PCI (*n*, %)	–	11 (8)	18 (23)	–	141 (94)	298 (95)	50 (100)	–	–
DES CVO PCI (*n*, %)	–	10 (12)	7 (17)	–	127 (87)	290 (93)	50 (100)	–	–
Anterior MI MV PCI (*n*, %)	27 (52)	59 (45)	17 (21)	67 (29)	54 (36)	105 (33)	24^b^ (48)	105 (36)	661^b^ (33)
Anterior MI CVO PCI (*n*, %)	10 (59)	35 (42)	12 (29)	89 (39)	52 (36)	112 (36)	23^b^ (46)	206 (35)	656^b^ (32)
2 VD MV PCI (*n*, %)	36 (69)	82 (63)	60 (75)	143 (61)	119 (79)	217 (69)	36 (72)	204 (69)	1453 (72)
2 VD CVO PCI (*n*, %)	9 (53)	63 (75)	33 (80)	155 (67)	110 (75)	213 (68)	33 (66)	396 (67)	1485 (73)
3 VD MV PCI (*n*, %)	16 (31)	48 (37)	20 (25)	91 (39)	31 (21)	97 (31)	14 (28)	91 (31)	452 (22)
3 VD CVO PCI (*n*, %)	8 (47)	21 (25)	8 (20)	76 (33)	36 (25)	100 (32)	17 (34)	194 (33)	436 (22)

*CVO PCI* culprit vessel-only percutaneous coronary intervention, *MV PCI* multivessel PCI, *DES* drug eluting stent, *IQR* interquartile range, *MI* myocardial infarction, *SD* standard deviation, *VD* vessel disease, – not reported

^a^Only the combination of ASA + clopidogrel/ticagrelor/prasugrel is reported

^b^Retrospectively defined as culprit-lesion located in the left anterior descending or left-main coronary artery

^c^Mean; SD not reported

^d^No differentiation made between clopidogrel, ticagrelor and prasugrel

### Outcome analysis

**Fig. 2:**
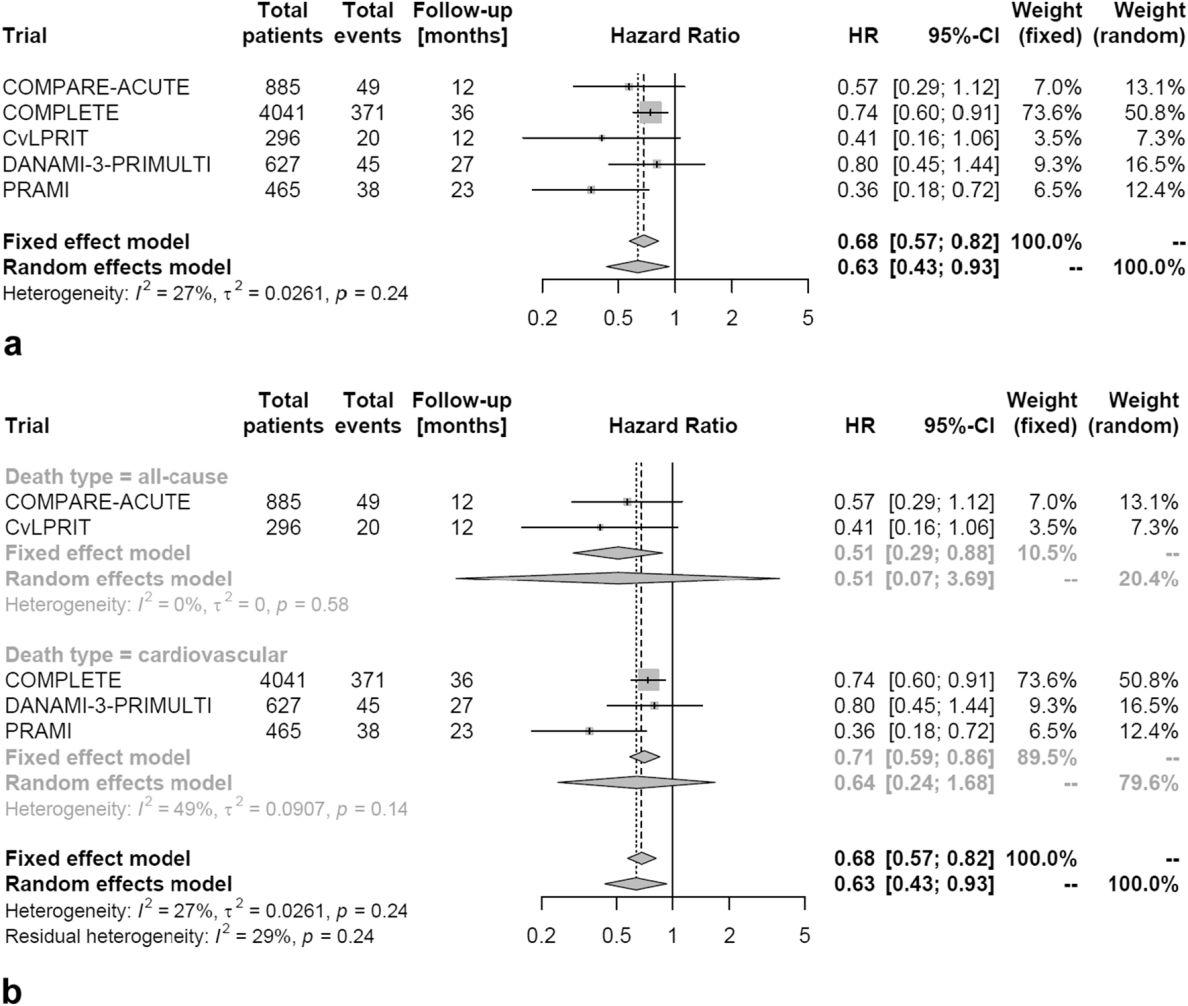
Occurrence of the combined primary endpoint according to revascularization strategy (multivessel PCI vs. culprit vessel-only PCI). **a** Combination of death (all-cause or cardiovascular death) and myocardial reinfarction. **b** After stratification for cause of death. *HR* hazard ratio

**Fig. 3:**
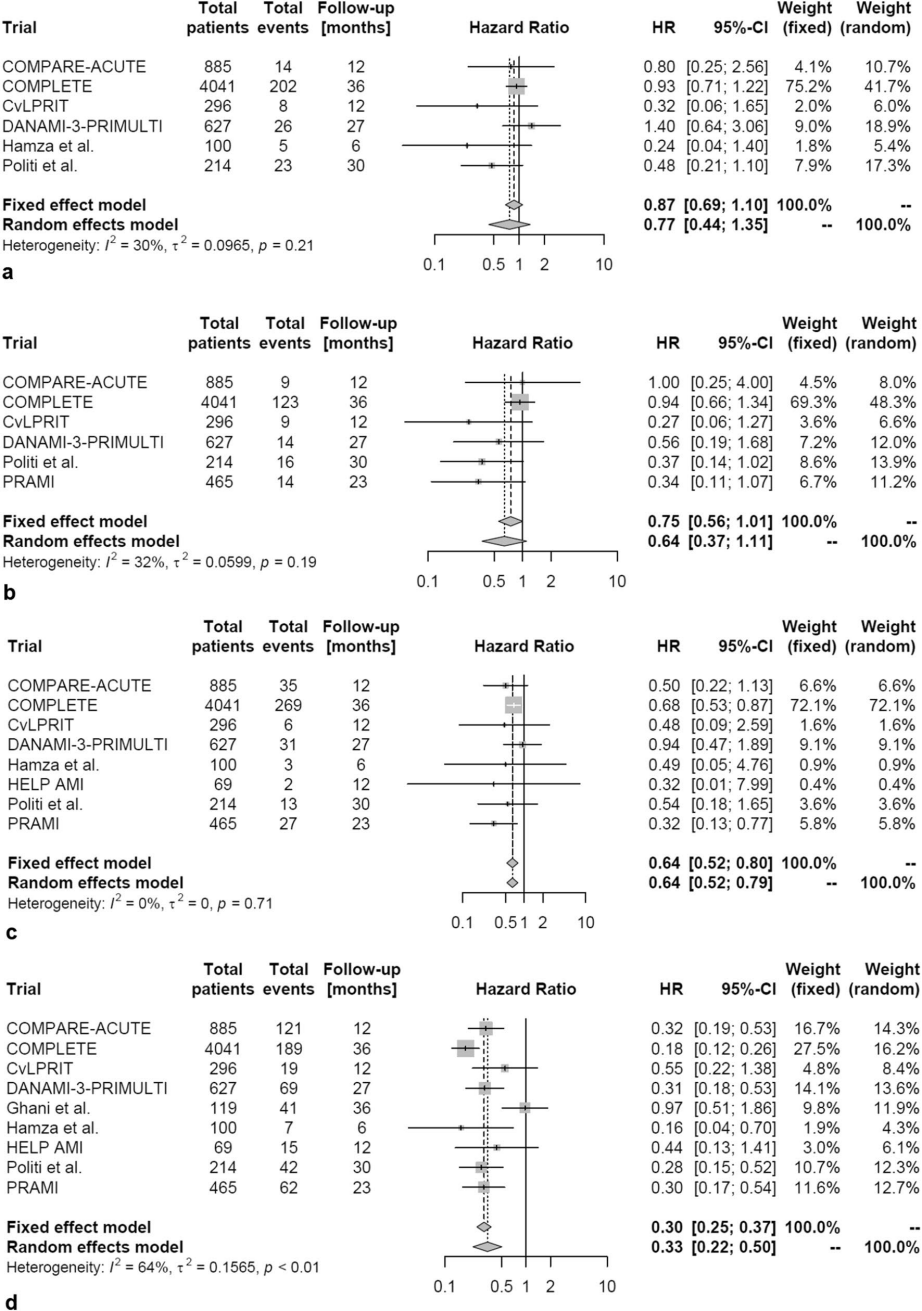
Occurrence of single endpoints according to revascularization strategy (multivessel PCI vs. culprit vessel-only PCI). **a** All-cause death; **b** cardiovascular death; **c** reinfarction; **d** repeat revascularization. *HR* hazard ratio

**Fig. 4:**
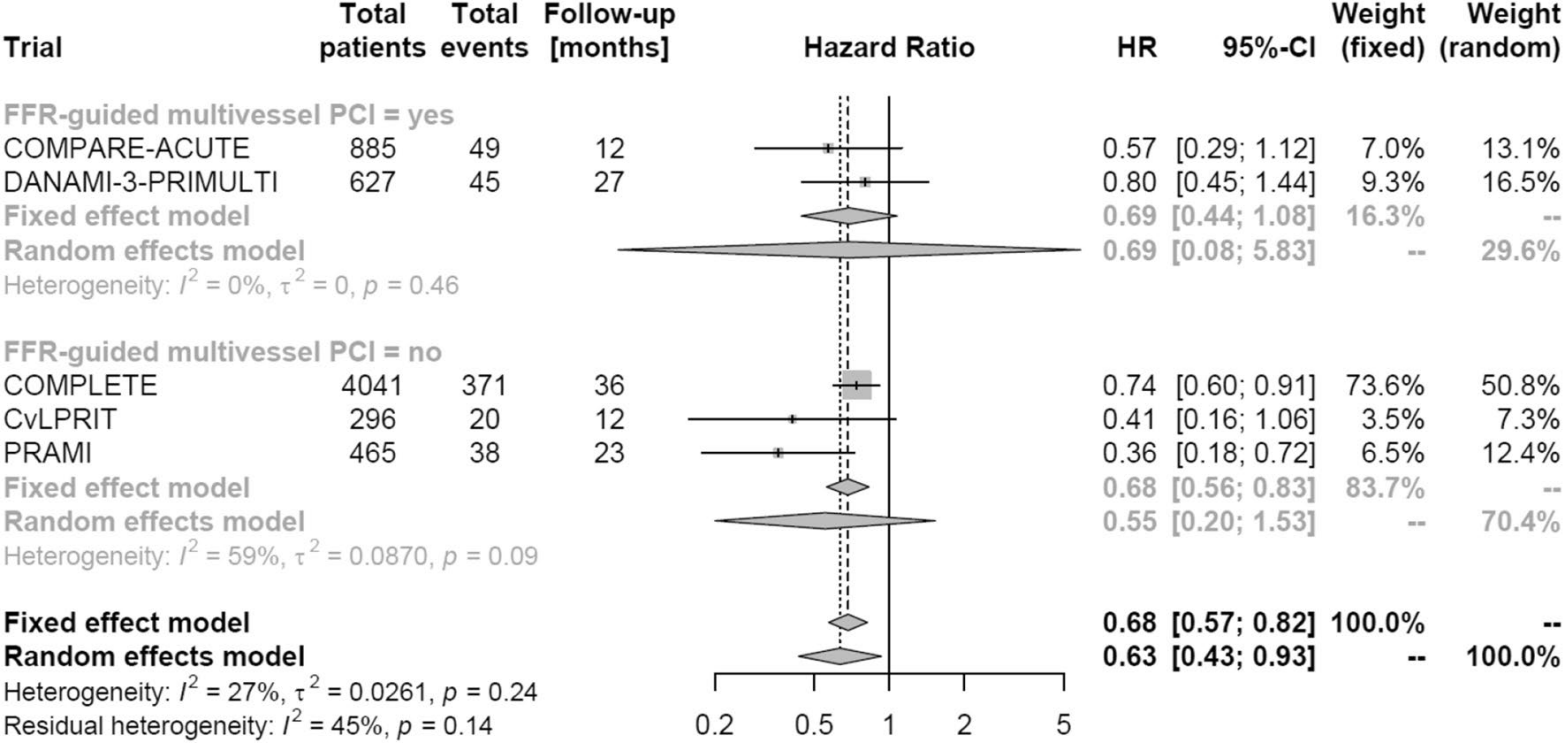
Occurrence of the combined primary endpoint after stratification for FFR-guided vs. angiography-guided multivessel PCI. *FFR* fractional flow reserve, *HR* hazard ratio, *PCI* percutaneous coronary intervention

In total, 523 (8.3%) of 6314 patients suffered the combined endpoint of death or non-fatal myocardial reinfarction. In patients treated with multivessel PCI this occurred less often than in patients treated by culprit vessel-only PCI (HR 0.63, 95% confidence interval [CI] 0.43–0.93; *p* = 0.03; Fig. 2a). According to stratified analysis, the reduction of the primary endpoint was not related to the mode of death reported in the individual trial (i.e. all-cause death versus cardiovascular death combined with non-fatal myocardial reinfarction; *p* value for between-group difference = 0.40; Fig. 2b). Looking on single endpoints, multivessel PCI did not reduce all-cause (HR 0.77, 95% CI 0.44–1.35, *p* = 0.28; Fig. 3a) or cardiovascular mortality (HR 0.64, 95% CI 0.37–1.11, *p* = 0.09; Fig. 3b) in comparison to culprit vessel-only PCI. However, the risks of non-fatal myocardial reinfarction (HR 0.64, 95% CI 0.52–0.79; *p* = 0.001; Fig. 3c) and repeat revascularization (HR 0.33, 95% CI 0.22–0.50, *p* < 0.001; Fig. 3d) were reduced with multivessel PCI compared to culprit vessel-only PCI. In a stratified analysis, the use of FFR-guided multivessel PCI (HR 0.69, 95% CI 0.08–5.83) did not affect the risk for the combined endpoint of death or non-fatal myocardial reinfarction compared to an angiography-guided strategy (HR 0.55, 95% CI 0.20–1.53; *p* value for between-group difference = 0.44; Fig. 4). Moreover, heterogeneity within the overall analysis was not explained by FFR versus no FFR strategy (*I*^2^ = 27% in the overall population versus *I*^2^ = 0% in FFR-guided and *I*^2^ = 59% in non-FFR-guided trials).

The benefit of multivessel PCI regarding the primary endpoint was formally greater in trials performing multivessel PCI predominantly within the index procedure as compared to trials of multivessel PCI in a staged procedure (HR 0.45, 95% CI 0.24–0.84 versus HR 0.75, 95% CI 0.55–1.02; *p* value for between-group difference < 0.001). This interaction was also present in the fixed effect model (*p* value for between-group difference = 0.03). This stratification abandoned heterogeneity (*I*^2^ = 27% in the overall population versus *I*^2^ = 0% in both strata). Visual inspection did not suggest small-study effects for any of the performed meta-analyses (Online Supplement Section 6, Figs. 2–6).

## Discussion

In the present meta-analysis, multivessel revascularization by PCI was associated with a significant reduction of the combined endpoint of death and non-fatal myocardial reinfarction in STEMI patients without cardiogenic shock. This finding was driven by a reduction of non-fatal myocardial reinfarction in the multivessel PCI group, whereas the rates of all-cause and cardiovascular death were similar between groups. As expected, repeat revascularizations were also significantly reduced in patients receiving multivessel PCI. FFR-guided revascularization did not impact prognosis compared to an angiography-guided approach.

Early revascularization of the infarct-related artery is crucial to reduce myocardial damage and preserve myocardial function and the detrimental effects of treatment delay have been shown in many studies [17, 18]. Single-stage multivessel PCI in STEMI patients without cardiogenic shock was not recommended in earlier STEMI guidelines based on adverse outcome with multivessel PCI reported by observational studies [4, 19–21]. However, observational studies are prone for significant selection bias since patients with more severe atherosclerotic lesions in non-culprit vessels might more likely receive multivessel PCI. During the last years several RCTs were conducted, consistently showing a benefit with multivessel PCI [6–9]. These findings were substantially driven by a reduction of subsequent revascularizations in most of these trials [8, 9].

The recently published COMPLETE trial reported a reduction of the combined endpoint of cardiovascular death or non-fatal myocardial infarction [10]. Since COMPLETE is the by far largest trial in the field, the results of the present meta-analysis are substantially driven by this trial. Even though COMPLETE’s weight is higher in fixed effect meta-analyses as compared to random-effects meta-analyses, visual inspection of funnel plots did not indicate asymmetry and hence small-study effects.

Another recently published meta-analysis which also included the data of the COMPLETE trial showed, in contrast to our meta-analysis, a significant reduction of cardiovascular mortality with complete revascularization [22]. This discrepancy results from different statistical approaches. We pre-specified in our PROSPERO registration (CRD42019142643) that we will estimate the between-study variance according to Paule–Mandel with Hartung–Knapp adjustment. The above-mentioned meta-analysis by Pavasini et al. used the DerSimonian–Laird estimator without Hartung–Knapp adjustment for estimating the between-study variance in their random-effects meta-analysis. It is well known that the DerSimonian–Laird estimator is prone to produce false positive findings especially in case when the number of pooled trials is small. The findings reported by Pavasini et al. should therefore be interpreted with caution. In line with our findings, the by far largest and well powered COMPLETE trial did not show any difference in cardiovascular mortality.

The COMPLETE trial as well as the present meta-analysis demonstrate that the benefits of multivessel PCI are attributed to a reduction of myocardial reinfarction. COMPLETE indicates that multivessel PCI after STEMI can prevent future coronary events, irrespective of the presence of objective or subjective signs of myocardial ischemia. One might speculate, how far these findings will impact future recommendations regarding multivessel PCI in STEMI patients.

The finding that multivessel PCI is more beneficial when performed within the index procedure should be interpreted with caution [23–25]. Perhaps, detection bias could serve as an explanation, as periprocedural infarctions might be less frequently diagnosed in patients receiving immediate multivessel PCI, since new troponin elevations or ECG changes might be incorrectly attributed to the index infarction. Importantly, periprocedural infarction was not well defined in several included trials (Online Supplement Section 3). Better outcome with immediate multivessel PCI might be explained by a higher 30-day mortality in STEMI patients with additional stenoses in non-culprit coronary arteries compared to those without additional stenoses [1]. On the other hand, the number of patients randomized during off hours is unclear from the trial reports. It might be possible that multivessel PCI is only beneficial during routine practice when maximal technical support and infrastructure is available [26]. Otherwise, multivessel PCI during off hours could be even harmful in some cases. This consideration might have influenced the COMPLETE study protocol, now leading to the situation that data for staged multivessel PCI is most robust.

The use of FFR-guided multivessel PCI was not associated with the risk for death or reinfarction compared to an angiography-guided approach. According to two RCTs, FFR-guided multivessel PCI could neither reduce mortality nor the rate of reinfarctions [8, 9]. In our meta-analysis comparison of FFR- and angiography-guided multivessel PCI is hampered by the diverse definition of FFR- and diameter stenosis-thresholds among the included trials (Table 1). However, the limited number of included trials hampers separate outcome analysis for different cut-off values from a statistical point of view.

The risk of bias was substantial in four trials, which all comprised a lower number of included patients compared to the five trials with a low risk of bias [6–9, 16, 27–29]. Nevertheless, we could not detect any substantial small-study effects by visual estimation.

### Limitations

In contrast to most previous meta-analyses, our meta-analysis took differences in length of follow-up in consideration. However, it is only based on published aggregated data. Unfortunately, not all eligible trials reported HRs with respective 95% CI. We therefore had to estimate these measures. Our approach is mathematically very similar to the approach used by Bangalore et al. [23]. Since the weight of the trials with estimated HRs is rather low, the error introduced in our pooled estimate is most likely small. We did not include the trial of Politi et al. in our meta-analysis regarding death or non-fatal myocardial reinfarction. This endpoint was not reported and we believe that summing up event rates for death and non-fatal myocardial reinfarction should not be done. Such an approach is prone to error if the same patients had a non-fatal myocardial reinfarction event before they die. To assure a detailed risk of bias assessment, only trials with available full-text publication were included in our meta-analysis. Therefore, PRAGUE-13 was not considered [15]. However, PRAGUE-13 would account for only 3% of patients in our meta-analysis.

## Conclusion

Multivessel PCI in STEMI patients without cardiogenic shock significantly reduced the risk of death or non-fatal myocardial reinfarction compared to culprit vessel-only PCI. This finding was mainly driven by a reduced rate of reinfarction. FFR-guided multivessel PCI resulted in similar outcome compared to an angiography-guided approach.

## Electronic supplementary material

Below is the link to the electronic supplementary material.

Supplementary file1 (DOCX 601 kb)
