# Supplementary material for: Multivessel versus culprit-only PCI in STEMI patients with multivessel disease: meta-analysis of randomized controlled trials
Source: Clin Res Cardiol. 2020 Apr 1;109(11):1381–91. doi: 10.1007/s00392-020-01637-6 (PMC7588388; doi:10.1007/s00392-020-01637-6)
Supplement: Supplementary file 1 — Supplementary file1 (DOCX 601 kb) [file 392_2020_1637_MOESM1_ESM.docx]

**1. Search Strategy**

**PubMed Search**

("STEMI"[Title/Abstract] OR "ST-elevation myocardial infarction"[Title/Abstract] OR "ST-segment elevation myocardial infarction"[Title/Abstract] OR "ST elevation myocardial infarction"[Title/Abstract] OR "ST segment elevation myocardial infarction"[Title/Abstract] OR "ST elevation"[Title/Abstract])

AND

("multivessel"[Title/Abstract] OR "multi vessel"[Title/Abstract] OR "multi-vessel"[Title/Abstract] OR "complete"[Title/Abstract] OR "culprit"[Title/Abstract])

AND

("PCI"[Title/Abstract] OR "PPCI"[Title/Abstract] OR "pPCI"[Title/Abstract] OR "primary PCI"[Title/Abstract] OR "percutaneous coronary intervention"[Title/Abstract] OR "primary percutaneous coronary intervention"[Title/Abstract] OR "revascularization"[Title/Abstract] OR "revascularisation"[Title/Abstract] OR "angioplasty"[Title/Abstract])

AND

("randomized"[Title/Abstract] OR "randomised"[Title/Abstract] OR "random"[Title/Abstract] OR "randomly"[Title/Abstract])

**CENTRAL Search**

#1 STEMI

#2 ST-elevation myocardial infarction

#3 ST-segment elevation myocardial infarction

#4 ST elevation myocardial infarction

#5 ST segment elevation myocardial infarction

#6 ST elevation

#7 {or #1 - #6}

#8 multivessel

#9 multi vessel

#10 multi-vessel

#11 complete

#12 culprit

#13 {or #8 - #12}

#14 PCI

#15 PPCI

#16 pPCI

#17 primary PCI

#18 percutaneous coronary intervention

#19 primary percutaneous coronary intervention

#20 revascularization

#21 revascularisation

#22 angioplasty

#23 {or #14 - #22}

#24 randomized

#25 randomised

#26 random

#27 randomly

#28 {or #24 - #27}

#29 #7 and #13 and #23 and #28

**EMBASE Search**

1 (STEMI or "ST-elevation myocardial infarction" or "ST-segment elevation myocardial infarction" or "ST elevation myocardial infarction" or "ST segment elevation myocardial infarction" or "ST elevation").ab,ti. (43119)

2 (multivessel or "multi vessel" or "multi-vessel" or "complete" or "culprit").ab,ti. (1016944)

3 (PCI or "PPCI" or "pPCI" or "primary PCI" or "primary percutaneous coronary intervention" or "revascularization" or "revascularisation" or "angioplasty").ab,ti. (174029)

4 (randomized or "randomised" or "random" or "randomly").ab,ti. (1427274)

5 1 and 2 and 3 and 4 (802)

**2. Comparison of estimated *lnHR* with published *lnHR***

**
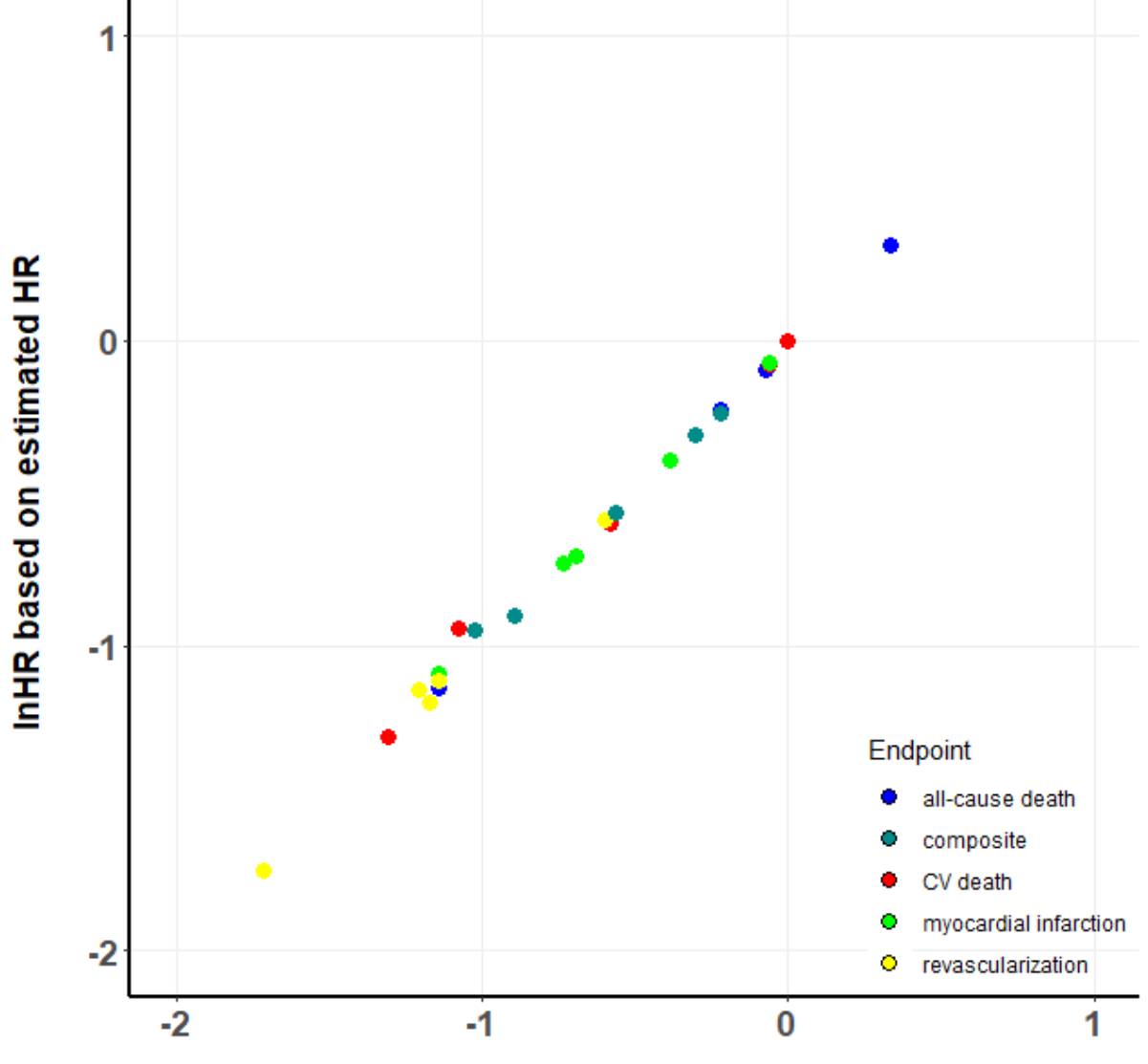
**

**Figure 1:** Scatter plot comparing the natural logarithm of the hazard ratio (lnHR) published in the original article with the lnHR estimated based on outcome events, number of patients, and mean follow-up time as described in the methods section in more details. This comparison was possible for the following studies which published HRs: COMPARE-ACUTE, COMPLETE, CvLPRIT, DANAMI-3-PRIMULTI, and PRAMI.

CV = cardiovascular, HR = hazard ratio.

**3. Definition of Study Endpoints**

|  | **Myocardial Reinfarction** | **Revascularization** |
| --- | --- | --- |
| HELP AMI (1) | n.r. | n.r. |
| Politi et al. (2) | n.r. | All PCI or CABG occurring after the baseline procedure and justified by recurrent symptoms, reinfarction or objective evidence of significant ischemia on provocative testing. Among repeat PCI, staged procedures already scheduled were excluded. In the staged group only unplanned procedures were classified as revascularization. |
| Ghani et al. (3) | New Q waves on the ECG or a new creatine kinase (CK) and CK-MB rise above the upper limit of normal. Periprocedural infarctions were included. | n.r. |
| PRAMI (4) | Symptoms of cardiac ischemia and a troponin level above the 99^th^ percentile. For patients with a recurrent myocardial infarction within 14 days after randomization,  the definition required new electrocardiographic evidence of ST-segment elevation or left bundle-branch block and angiographic evidence of coronary-  artery occlusion. | Repeat revascularization procedures (PCI or CABG). |
| CvLPRIT (5) | One or more of the following required: Type 1: Spontaneous re-MI: Recurrent angina symptoms or new ECG changes occurring before PCI or <48 hours from PCI that is compatible with re-MI associated with an elevation of CK-MB, troponin, or total CK beyond ULN and 20% or more above the previous value. Type 4a: CK-MB or total CK >3 times the ULN within 48 hours following PCI. If the pre-PCI CK-MB or total CK level is higher than the ULN, there also needs to be:  either the demonstration of a falling CK-MB or total CK level prior to the onset of the suspected event, or a subsequent peak of the cardiac biomarker of at least 20% above the previous value obtained prior to the onset of the suspected event.  With either an appropriate clinical presentation or new ischemic ECG changes (ST-segment depression or ST-segment elevation or development of new pathological Q waves/LBBB).  Type 4b: Myocardial infarction associated with stent thrombosis as documented by angiography or at autopsy AND fulfilling the criteria of spontaneous MI (Type 1). | Repeat revascularizations classified as:  1. Target lesion re-interventions (TLR) inside the implanted stent or within 5 mm proximally or distally or repeated interventions in the same vessel (TVR) by percutaneous coronary interventions (PCI) or by coronary artery bypass graft surgery.  2. PCI to lesions not identified previously  3. CABG for new symptoms or complications of PCI. |
| DANAMI-3-PRIMULTI (6) | Typical chest pain accompanied by a substantial rise in troponins, development of new Q-waves on the electrocardiograph, or both. | Ischemia-driven revascularization (subjective or objective) of lesions in non-infarct-related arteries. |
| Hamza et al. (7) | n.r. | Ischemia-driven revascularization by PCI or CABG. |
| COMPARE-ACUTE (8) | n.r. | Defined as any revascularization. |
| COMPLETE (9) | The 3^rd^ universal definition of myocardial infarction, with modification to the ACS setting, will be used. Any one of the following criteria meets the diagnosis for myocardial infarction:  1.  a) For individuals with normal (or presumed normal) cardiac troponin values (cTn), detection of a rise and/or fall of cardiac troponin values with at least one value above the 99th percentile upper reference limit (URL) and with at least one of the following:   - New symptoms suggestive of myocardial ischemia (i.e., significant, sustained ischemic symptoms lasting at least 20 minutes) or - New or presumed new significant ST–T wave changes or new left bundle branch block (LBBB) - Development of pathological Q waves on the ECG - Imaging evidence of new loss of viable myocardium or new regional wall motion abnormality - Identification of an intracoronary thrombus by angiography or autopsy   b) For individuals whose cardiac troponin values are already elevated or were recently elevated (i.e., suspected new MI occurs within 1 week of the last troponin measurement), new ischemic symptoms of at least 20 minutes and either new ST-segment elevation of at least 1 mm in 2 adjacent limb leads or 2 mm in 2 adjacent precordial leads are required. These ECG changes must be distinct from the original MI and not due to the usual ECG evolution of this event.  2.  Cardiac death with symptoms suggestive of myocardial ischemia and presumed new ischemic ECG changes or new LBBB, but death occurred before the cardiac troponin measurements were obtained, or before cardiac troponin values would be increased.  3.  Possible MI in conjunction with PCI:  a) For individuals with normal or presumed normal cardiac troponin prior to percutaneous coronary intervention (PCI), a PCI-related MI within 24 hours following PCI is defined by elevation of cardiac troponin values (35X 99^th^ percentile URL or CK-MB>5xURL) and with at least one of the following:   - New symptoms suggestive of myocardial ischemia (i.e., significant, sustained ischemic symptoms lasting at least 20 minutes) or - New or presumed new significant ST–T wave changes or new left bundle branch block (LBBB) or - Angiographic findings consistent with a major procedural complication (e.g., abrupt closure, no reflow, new angiographic thrombus, distal embolization, major side branch occlusion or dissection with reduced flow) or - Imaging evidence of new loss of viable myocardium or new regional wall motion abnormality is required.   b) For individuals whose cardiac troponin values are already elevated or were recently elevated (i.e., suspected new MI occurs within 1 week of the last troponin measurement), new ischemic symptoms of at least 20 minutes and either new ST-segment elevation of at least 1 mm in 2 adjacent limb leads or 2 mm in 2 adjacent precordial leads are required. These ECG changes must be distinct from the original MI and not due to the usual ECG evolution of this event.  4.  Stent thrombosis associated with MI when detected by coronary angiography or autopsy in the setting of myocardial ischemia and with a rise and/or fall of cardiac troponin values with at least one value above the 99^th^ percentile URL.  Note: The incidental angiographic documentation of stent occlusion in the absence of clinical signs or symptoms is not considered a confirmed stent thrombosis related MI (silent occlusion).  5.  Coronary artery bypass grafting (CABG) related MI is defined by elevation of cardiac troponin values (>70 X 99^th^ percentile URL) in patients with normal baseline troponin values (>99^th^ percentile URL or CK-MB>10x URL) within 24 h of CABG) and with at least one of the following:   - New pathological Q waves or new LBBB, or - Angiographic documented new graft or new native coronary artery occlusion, or - Imaging evidence of new loss of viable myocardium or new regional wall motion abnormality. | Defined as ischemia-driven revascularization:  Requires all of the following criteria:   - 1. Ischemic symptoms consistent with CCS class ≥ 2 angina despite optimal medical therapy, and   2. PCI or CABG of either the culprit lesion (within 5 mm of the stented segment) associated with the index PCI or a non-culprit lesion that led to enrollment into the trial, and   3. At least ONE of the following: - Positive functional study (Exercise or Persantine myocardial perfusion imaging or stress or dobutamine echo or other imaging) demonstrating clear evidence of reversible ischemia corresponding to a stenosis - New Ischemic ECG changes at rest or with exertion in a distribution consistent with a stenosis - FFR ≤0.80 |

n.r. = not reported.

**4. Risk of Bias Assessment**

HELP AMI (1)

| **Domain** | **Support for judgement** | **Review authors’ judgement** |
| --- | --- | --- |
| **Risk of selection bias** |  |  |
| Random sequence generation | 3:1 randomization to complete revascularization and culprit vessel-only PCI. | Low risk of bias (+) |
| Allocation concealment | 3:1 randomization to complete revascularization and culprit vessel-only PCI. | Low risk of bias (+) |
| **Performance bias** |  |  |
| Blinding of participants and personnel. Assessments should be made for each main outcome (or class of outcomes). | Patients and personnel were not blinded to the assigned treatment. | High risk of bias (+) |
| **Detection bias** |  |  |
| Blinding of outcome assessment. Assessments should be made for each main outcome (or class of outcomes). | It was not reported whether outcomes were adjudicated by an independent clinical events committee or not. | High risk of bias (+) |
| **Attrition bias** |  |  |
| Incomplete outcome data. Assessments should be made for each main outcome (or class of outcomes). | It was not reported whether outcomes were analyzed according to an intention-to-treat protocol. | High risk of bias (+) |
| **Reporting bias** |  |  |
| Selective reporting | The reported endpoints were not pre-specified. | High risk of bias (+) |

Politi et al. (2)

| **Domain** | **Support for judgement** | **Review authors’ judgement** |
| --- | --- | --- |
| **Risk of selection bias** |  |  |
| Random sequence generation | Patients were randomly allocated to three different treatment strategies (computer-based randomization). | Low risk of bias (+) |
| Allocation concealment | Patients were randomly allocated to three different treatment strategies using (computer- based randomization). | Low risk of bias (+) |
| **Performance bias** |  |  |
| Blinding of participants and personnel. Assessments should be made for each main outcome (or class of outcomes). | Patients and personnel were not blinded to the assigned treatment. | High risk of bias (+) |
| **Detection bias** |  |  |
| Blinding of outcome assessment. Assessments should be made for each main outcome (or class of outcomes). | It was not reported whether outcomes were adjudicated by an independent clinical events committee or not. | High risk of bias (+) |
| **Attrition bias** |  |  |
| Incomplete outcome data. Assessments should be made for each main outcome (or class of outcomes). | Data were analyzed as intention-to-treat. | Low risk of bias (+) |
| **Reporting bias** |  |  |
| Selective reporting | The reported endpoints were not pre-specified. | High risk of bias (+) |

Ghani et al. (3)

| **Domain** | **Support for judgement** | **Review authors’ judgement** |
| --- | --- | --- |
| **Risk of selection bias** |  |  |
| Random sequence generation | Randomisation was performed by means of a computer program. Patients were allocated to an invasive or conservative treatment in a 2:1 ratio respectively. | Low risk of bias (+) |
| Allocation concealment | Randomisation was performed by means of a computer program. Patients were allocated to an invasive or conservative treatment in a 2:1 ratio respectively. | Low risk of bias (+) |
| **Performance bias** |  |  |
| Blinding of participants and personnel. Assessments should be made for each main outcome (or class of outcomes). | Patients and personnel were not blinded to the assigned treatment. | High risk of bias (+) |
| **Detection bias** |  |  |
| Blinding of outcome assessment. Assessments should be made for each main outcome (or class of outcomes). | It was not reported whether outcomes were adjudicated by an independent clinical events committee or not. | High risk of bias (+) |
| **Attrition bias** |  |  |
| Incomplete outcome data. Assessments should be made for each main outcome (or class of outcomes). | Endpoints were analyzed according to an intention-to-treat protocol. | Low risk of bias (+) |
| **Reporting bias** |  |  |
| Selective reporting | The reported endpoints were not pre-specified. | High risk of bias (+) |

PRAMI (4)

| **Domain** | **Support for judgement** | **Review authors’ judgement** |
| --- | --- | --- |
| **Risk of selection bias** |  |  |
| Random sequence generation | After the completion of PCI in the infarct artery, eligible patients were randomly assigned to undergo no further PCI procedures or to undergo immediate preventive PCI in non-infarct arteries with more than 50% stenoses (preventive PCI). | Low risk of bias (+) |
| Allocation concealment | After the completion of PCI in the infarct artery, eligible patients were randomly assigned to undergo no further PCI procedures or to undergo immediate preventive PCI in non-infarct arteries with more than 50% stenoses (preventive PCI). | Low risk of bias (+) |
| **Performance bias** |  |  |
| Blinding of participants and personnel. Assessments should be made for each main outcome (or class of outcomes). | Patients and personnel were not blinded to the assigned treatment. | High risk of bias (+) |
| **Detection bias** |  |  |
| Blinding of outcome assessment. Assessments should be made for each main outcome (or class of outcomes). | An independent cardiologist and cardiac surgeon who were not notified about study-group assignments examined specified primary and secondary outcomes. These clinicians reviewed outcome events separately and then together for the five participating sites. | Low risk of bias (+) |
| **Attrition bias** |  |  |
| Incomplete outcome data. Assessments should be made for each main outcome (or class of outcomes). | Patients were analyzed according to the groups they were initially assigned to (intention-to-treat principle). | Low risk of bias (+) |
| **Reporting bias** |  |  |
| Selective reporting | The reported endpoints were pre-specified. Data regarding cardiac death, reinfarction, revascularization, and the combination of cardiac death or reinfarction were reported in the manuscript. | Low risk of bias (+) |

CvLPRIT (5)

| **Domain** | **Support for judgement** | **Review authors’ judgement** |
| --- | --- | --- |
| **Risk of selection bias** |  |  |
| Random sequence generation | Randomization was undertaken via a 24-h automated, voice-activated central system. | Low risk of bias (+) |
| Allocation concealment | Randomization was undertaken via a 24-h automated, voice-activated central system. | Low risk of bias (+) |
| **Performance bias** |  |  |
| Blinding of participants and personnel. Assessments should be made for each main outcome (or class of outcomes). | Patients and personnel were not blinded to the assigned treatment. | High risk of bias (+) |
| **Detection bias** |  |  |
| Blinding of outcome assessment. Assessments should be made for each main outcome (or class of outcomes). | Clinicians blinded to the randomization group  adjudicated all MACE and safety endpoints. | Low risk of bias (+) |
| **Attrition bias** |  |  |
| Incomplete outcome data. Assessments should be made for each main outcome (or class of outcomes). | The primary analysis was on an intention-to-treat basis of all randomized patients according to treatment group. | Low risk of bias (+) |
| **Reporting bias** |  |  |
| Selective reporting | The reported endpoints were pre-specified. Data regarding all-cause death, cardiac death, reinfarction, revascularization, and the combination of all-cause death or reinfarction were reported in the main manuscript or supplements. | Low risk of bias (+) |

DANAMI-3-PRIMULTI (6)

| **Domain** | **Support for judgement** | **Review authors’ judgement** |
| --- | --- | --- |
| **Risk of selection bias** |  |  |
| Random sequence generation | Randomisation was done with an electronic  web-based system in permuted blocks of varying size at each participating center. | Low risk of bias (+) |
| Allocation concealment | Randomisation was done with an electronic  web-based system in permuted blocks of varying size at each participating center. | Low risk of bias (+) |
| **Performance bias** |  |  |
| Blinding of participants and personnel. Assessments should be made for each main outcome (or class of outcomes). | Patients and personnel were not blinded to the assigned treatment. | High risk of bias (+) |
| **Detection bias** |  |  |
| Blinding of outcome assessment. Assessments should be made for each main outcome (or class of outcomes). | An independent data safety monitoring board supervised safety measurements and an independent clinical events committee adjudicated all events. | Low risk of bias (+) |
| **Attrition bias** |  |  |
| Incomplete outcome data. Assessments should be made for each main outcome (or class of outcomes). | Patients were analyzed according to the groups they were initially assigned to (intention-to-treat principle). | Low risk of bias (+) |
| **Reporting bias** |  |  |
| Selective reporting | The reported endpoints were pre-specified. Data regarding all-cause death, cardiac death, reinfarction, revascularization, and the combination of cardiac death or reinfarction were reported in the manuscript. | Low risk of bias (+) |

Hamza et al. (7)

| **Domain** | **Support for judgement** | **Review authors’ judgement** |
| --- | --- | --- |
| **Risk of selection bias** |  |  |
| Random sequence generation | 1:1 randomization was performed. | Low risk of bias (+) |
| Allocation concealment | 1:1 randomization was performed. | Low risk of bias (+) |
| **Performance bias** |  |  |
| Blinding of participants and personnel. Assessments should be made for each main outcome (or class of outcomes). | Patients and personnel were not blinded to the assigned treatment. | High risk of bias (+) |
| **Detection bias** |  |  |
| Blinding of outcome assessment. Assessments should be made for each main outcome (or class of outcomes). | It was not reported whether outcomes were adjudicated by an independent clinical events committee or not. | High risk of bias (+) |
| **Attrition bias** |  |  |
| Incomplete outcome data. Assessments should be made for each main outcome (or class of outcomes). | It was not reported whether outcomes were analyzed according to an intention-to-treat protocol. | High risk of bias (+) |
| **Reporting bias** |  |  |
| Selective reporting | The reported endpoints were not pre-specified. | High risk of bias (+) |

COMPARE-ACUTE (8)

| **Domain** | **Support for judgement** | **Review authors’ judgement** |
| --- | --- | --- |
| **Risk of selection bias** |  |  |
| Random sequence generation | Patients were randomly assigned in a ratio of 1:2 with the use of closed, opaque envelopes to FFR-guided complete revascularization or treatment of the infarct artery only. | Low risk of bias (+) |
| Allocation concealment | Patients were randomly assigned in a ratio of 1:2 with the use of closed, opaque envelopes to FFR-guided complete revascularization or treatment of the infarct artery only. | Low risk of bias (+) |
| **Performance bias** |  |  |
| Blinding of participants and personnel. Assessments should be made for each main outcome (or class of outcomes). | Patients and personnel were not blinded to the assigned treatment.  Patients in the infarct-artery-only group underwent the FFR procedure but were not aware of the findings. | High risk of bias (+) |
| **Detection bias** |  |  |
| Blinding of outcome assessment. Assessments should be made for each main outcome (or class of outcomes). | All events were analyzed and adjudicated by an independent clinical evaluation committee at a clinical research organization. | Low risk of bias (+) |
| **Attrition bias** |  |  |
| Incomplete outcome data. Assessments should be made for each main outcome (or class of outcomes). | Patients were analyzed according to the groups they were initially assigned to (intention-to-treat principle). | Low risk of bias (+) |
| **Reporting bias** |  |  |
| Selective reporting | The reported endpoints were pre-specified. Data regarding all-cause death, cardiac death, reinfarction, revascularization, and the combination of all-cause death or reinfarction were reported in the manuscript. | Low risk of bias (+) |

COMPLETE (9)

| **Domain** | **Support for judgement** | **Review authors’ judgement** |
| --- | --- | --- |
| **Risk of selection bias** |  |  |
| Random sequence generation | Randomization was performed as soon as possible (and no later than 72 hours) after culprit lesion index PCI, using a concealed, central, computerized randomization system. Randomization was performed as soon as possible (and no later than 72 hours) after culprit lesion index PCI, using a concealed, central, computerized randomization system. | Low risk of bias (+) |
| Allocation concealment | Randomization was performed as soon as possible (and no later than 72 hours) after culprit lesion index PCI, using a concealed, central, computerized randomization system. Randomization was performed as soon as possible (and no later than 72 hours) after culprit lesion index PCI, using a concealed, central, computerized randomization system. | Low risk of bias (+) |
| **Performance bias** |  |  |
| Blinding of participants and personnel. Assessments should be made for each main outcome (or class of outcomes). | Patients and personnel were not blinded to the assigned treatment. | High risk of bias (+) |
| **Detection bias** |  |  |
| Blinding of outcome assessment. Assessments should be made for each main outcome (or class of outcomes). | An Event Adjudication Committee, consisting of clinicians who were masked to treatment  allocation, adjudicated primary and secondary efficacy outcomes as well as bleeding events. | Low risk of bias (+) |
| **Attrition bias** |  |  |
| Incomplete outcome data. Assessments should be made for each main outcome (or class of outcomes). | All randomized patients were included in the analysis according to the treatment group to which they were assigned, regardless of the treatment they actually received (i.e., intention-to-treat principle). | Low risk of bias (+) |
| **Reporting bias** |  |  |
| Selective reporting | The reported endpoints were pre-specified. Data regarding all-cause death, cardiovascular death, reinfarction, revascularization, and the combination of cardiovascular death or reinfarction were reported in the main manuscript. | Low risk of bias (+) |

**5. Quality of Included Trials**

| ITT analysis | n.r. | yes | yes | Yes | yes | yes | n.r. | yes | yes |
| --- | --- | --- | --- | --- | --- | --- | --- | --- | --- |
| Registration of trial | no | no | no | Yes | yes | yes | no | yes | yes |
| Mean^1^/median^2^ follow-up reported | no | yes^1^ | no | yes^1^ | yes^2^ | yes^2^ | no | no | yes^1^ |
| Number of crossovers reported | no | no | no | No | yes | yes | no | yes | yes |
|  | HELP AMI | Politi et al. | Ghani et al. | PRAMI | CvLPRIT | DANAMI-3-PRIMULTI | Hamza et al. | COMPARE-ACUTE | COMPLETE |

n.r. = not reported.

**6. Funnel Plots**


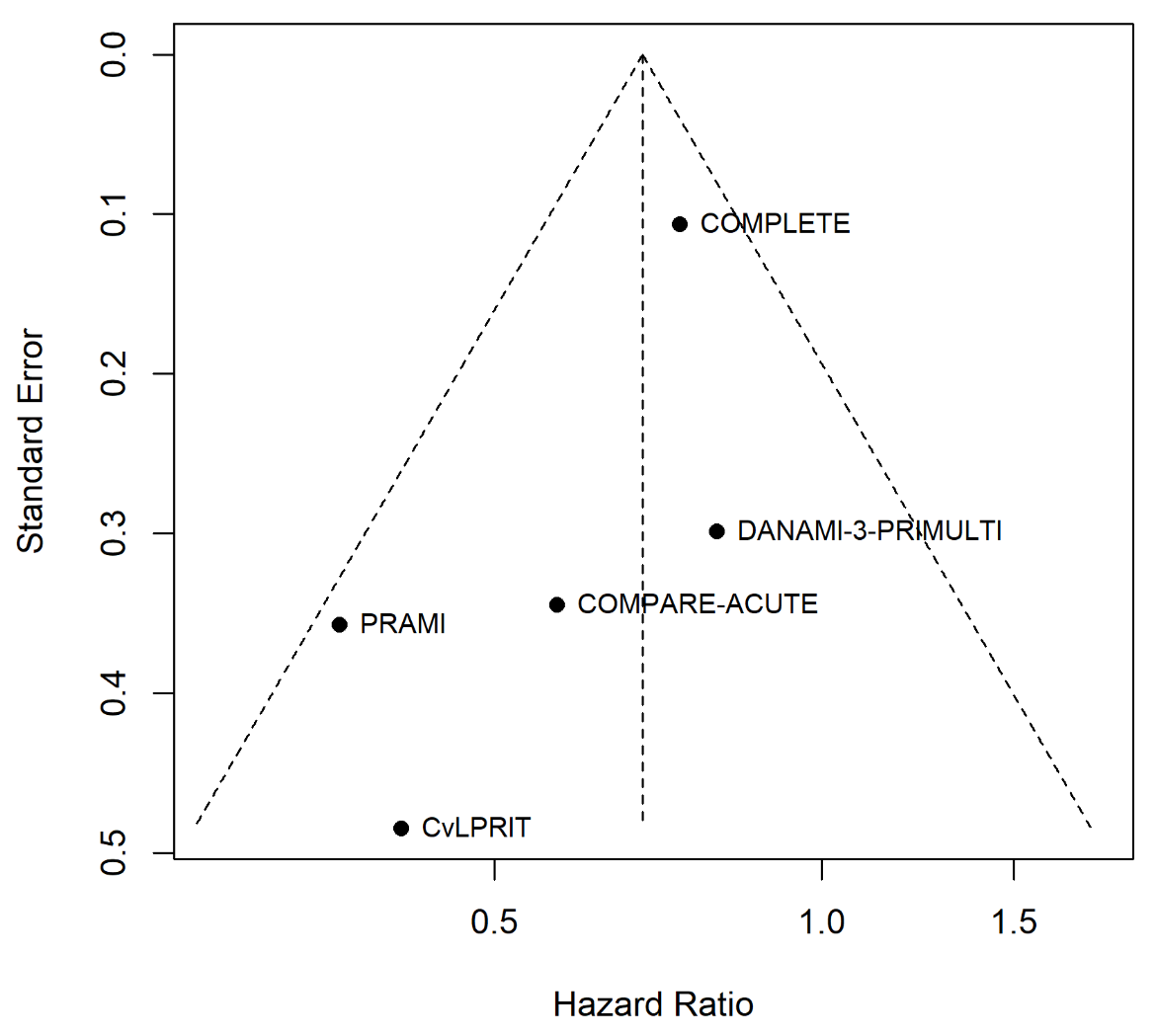


**Figure 2: Funnel plot for the composite of death or non-fatal myocardial infarction.** Plotted are the hazard ratio on the x-axis versus the standard error of its natural logarithm on the y-axis. The triangle is centered on the pooled fixed effects estimate (vertical line) and extends to the corresponding confidence limits.


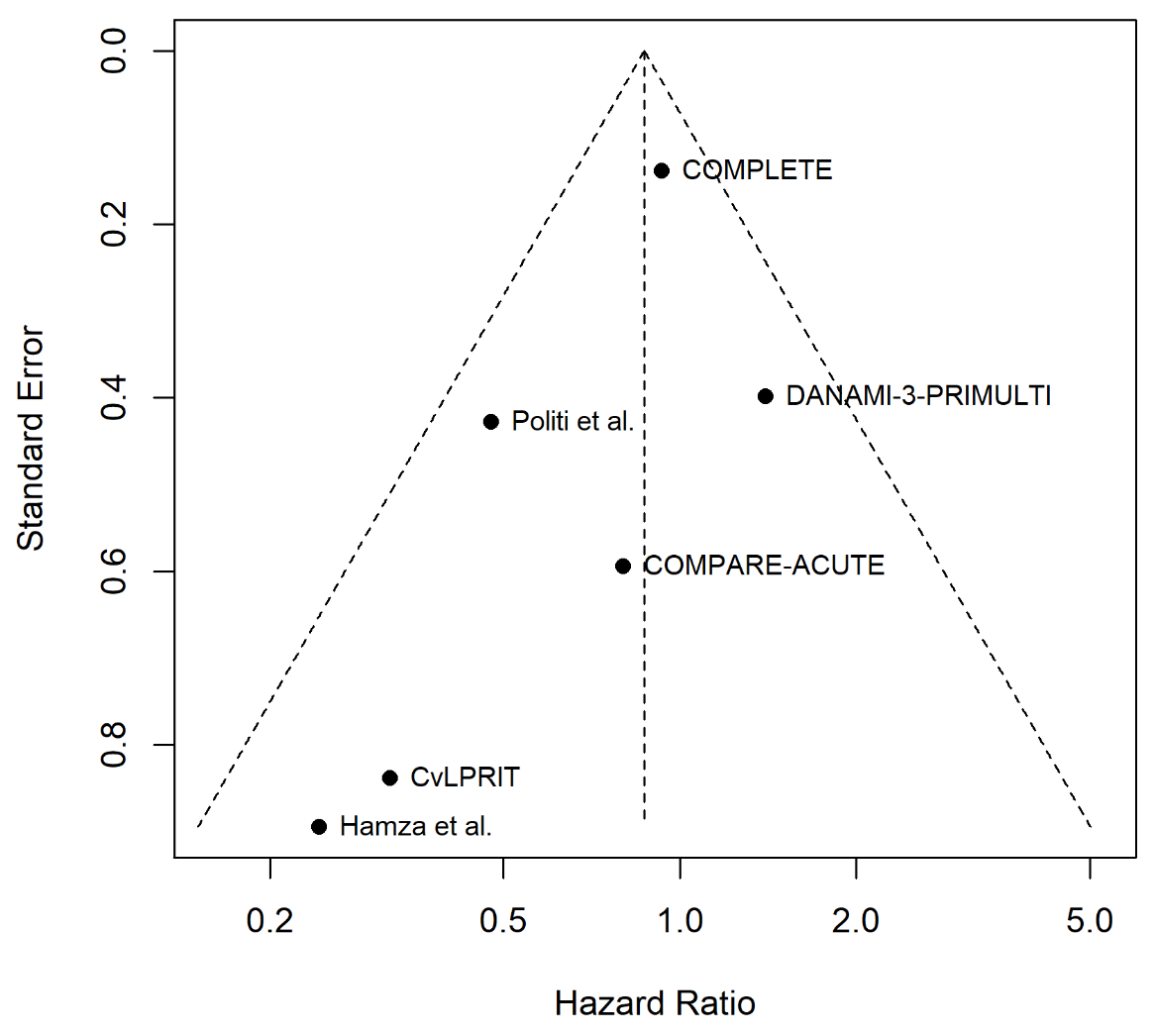


**Figure 3: Funnel plot for all-cause death.** Plotted are the hazard ratio on the x-axis versus the standard error of its natural logarithm on the y-axis. The triangle is centered on the pooled fixed effects estimate (vertical line) and extends to the corresponding confidence limits.


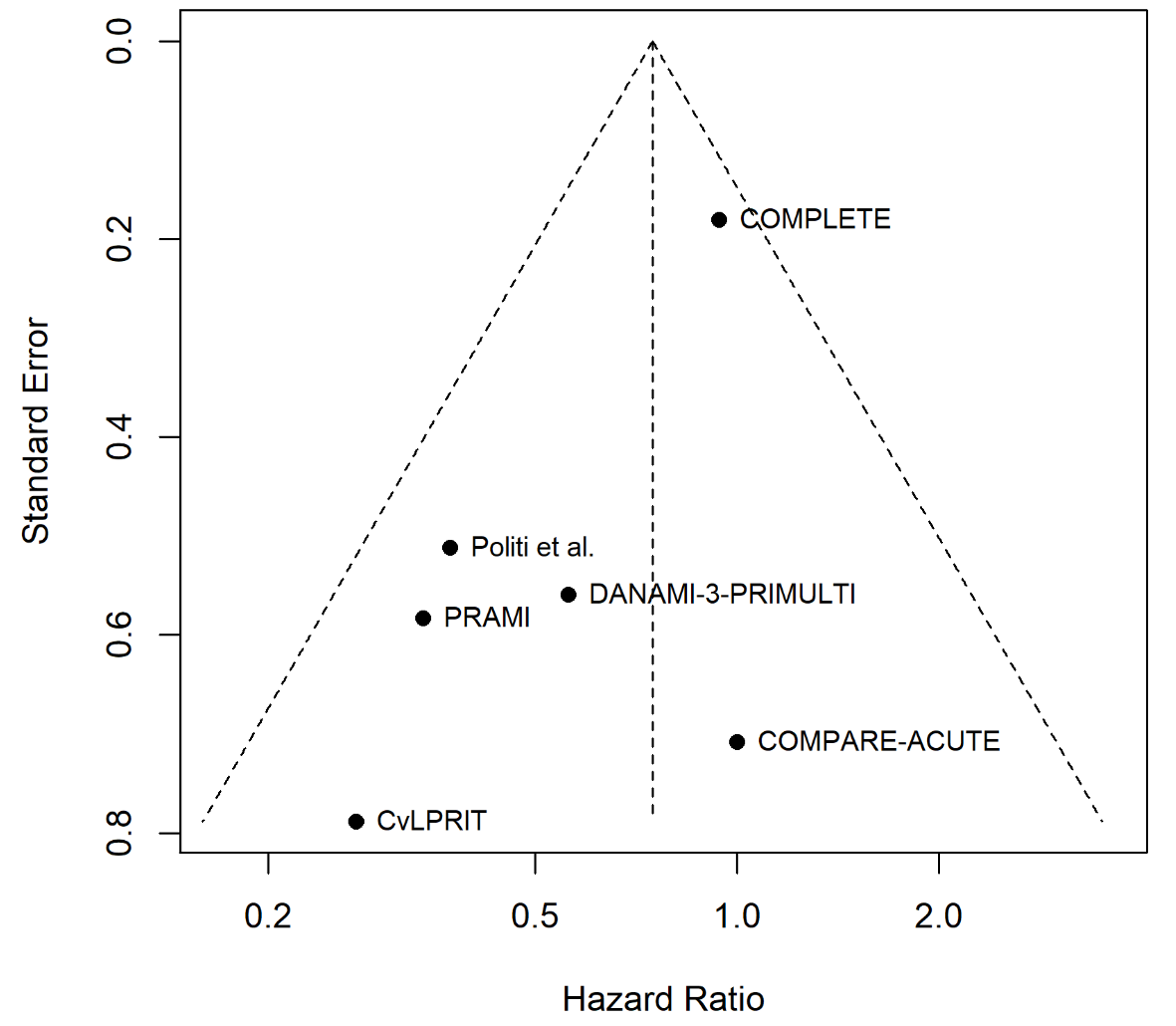


**Figure 4: Funnel plot for cardiovascular death.** Plotted are the hazard ratio on the x-axis versus the standard error of its natural logarithm on the y-axis. The triangle is centered on the pooled fixed effects estimate (vertical line) and extends to the corresponding confidence limits.


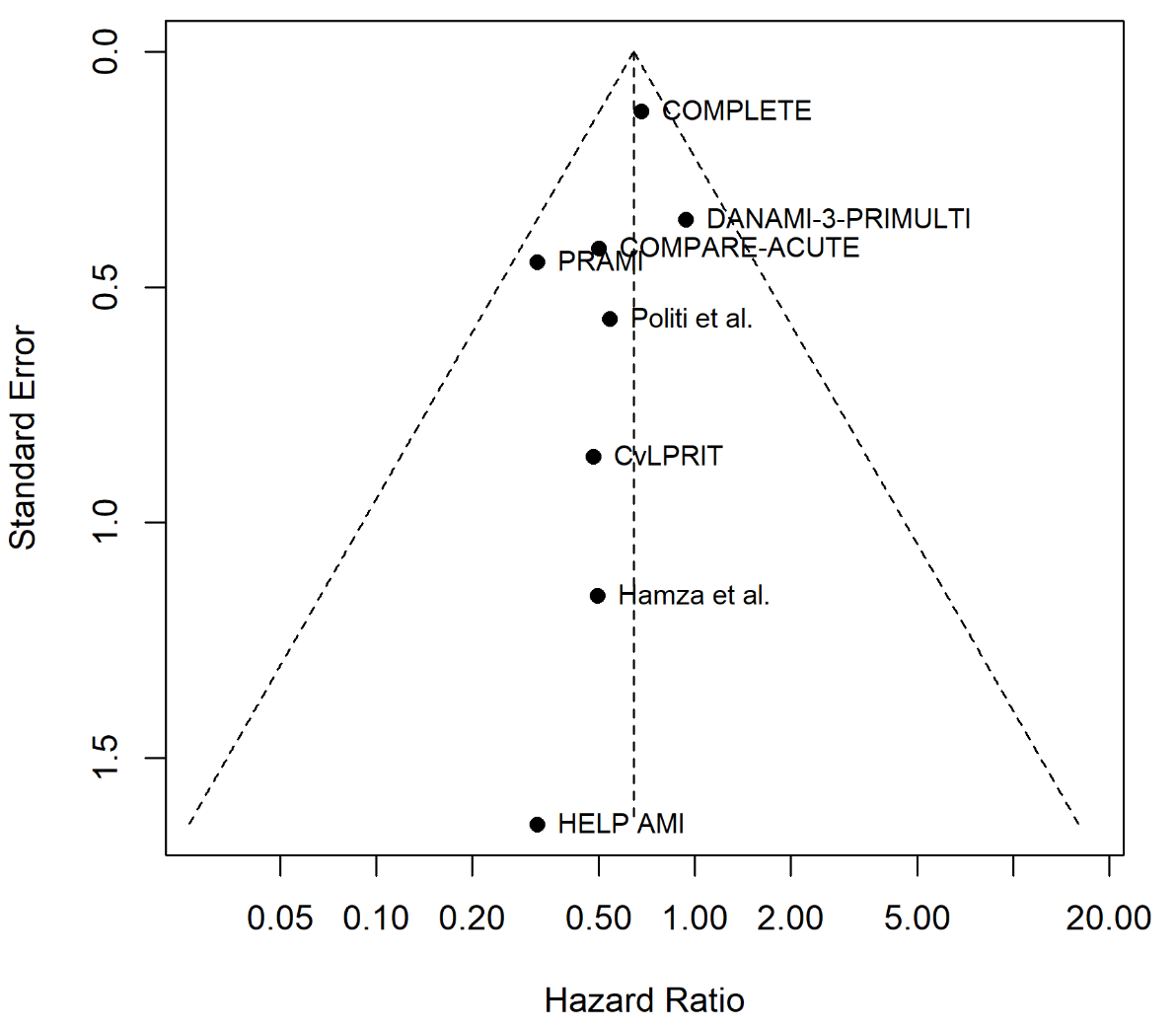


**Figure 5: Funnel pot for non-fatal myocardial infarction.** Plotted are the hazard ratio on the x-axis versus the standard error of its natural logarithm on the y-axis. The triangle is centered on the pooled fixed effects estimate (vertical line) and extends to the corresponding confidence limits.


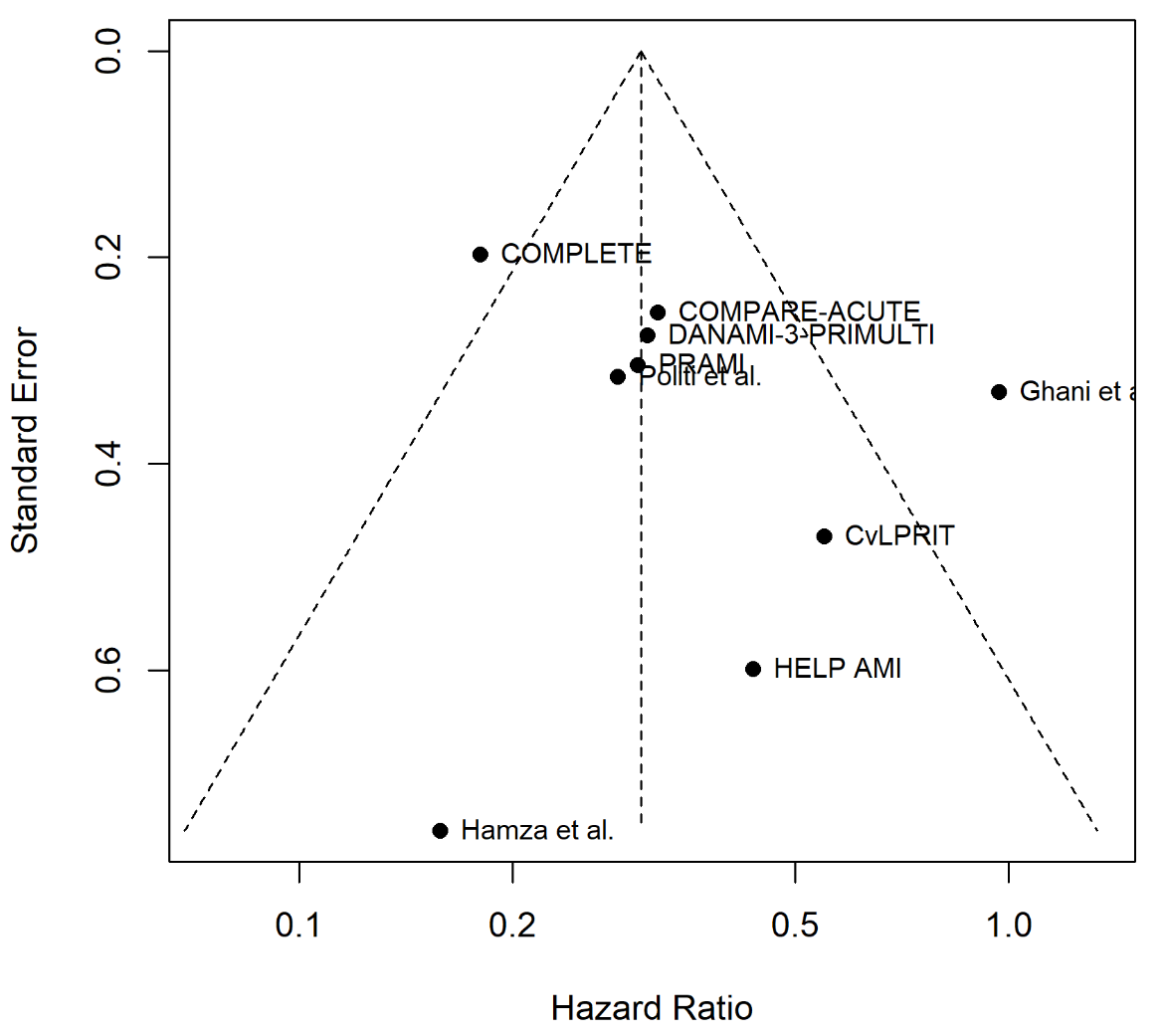


**Figure 6: Funnel plot for revascularization.** Plotted are the hazard ratio on the x-axis versus the standard error of its natural logarithm on the y-axis. The triangle is centered on the pooled fixed effects estimate (vertical line) and extends to the corresponding confidence limits.

**6. References**

1. Di Mario C, Mara S, Flavio A, Imad S, Antonio M, Anna P, et al. Single vs multivessel treatment during primary angioplasty: results of the multicentre randomised HEpacoat for cuLPrit or multivessel stenting for Acute Myocardial Infarction (HELP AMI) Study. International journal of cardiovascular interventions. 2004;6(3-4):128-33.

2. Politi L, Sgura F, Rossi R, Monopoli D, Guerri E, Leuzzi C, et al. A randomised trial of target-vessel versus multi-vessel revascularisation in ST-elevation myocardial infarction: major adverse cardiac events during long-term follow-up. Heart. 2010;96(9):662-7.

3. Ghani A, Dambrink JH, van 't Hof AW, Ottervanger JP, Gosselink AT, Hoorntje JC. Treatment of non-culprit lesions detected during primary PCI: long-term follow-up of a randomised clinical trial. Netherlands heart journal : monthly journal of the Netherlands Society of Cardiology and the Netherlands Heart Foundation. 2012;20(9):347-53.

4. Wald DS, Morris JK, Wald NJ, Chase AJ, Edwards RJ, Hughes LO, et al. Randomized trial of preventive angioplasty in myocardial infarction. N Engl J Med. 2013;369(12):1115-23.

5. Gershlick AH, Khan JN, Kelly DJ, Greenwood JP, Sasikaran T, Curzen N, et al. Randomized trial of complete versus lesion-only revascularization in patients undergoing primary percutaneous coronary intervention for STEMI and multivessel disease: the CvLPRIT trial. J Am Coll Cardiol. 2015;65(10):963-72.

6. Engstrom T, Kelbaek H, Helqvist S, Hofsten DE, Klovgaard L, Holmvang L, et al. Complete revascularisation versus treatment of the culprit lesion only in patients with ST-segment elevation myocardial infarction and multivessel disease (DANAMI-3-PRIMULTI): an open-label, randomised controlled trial. Lancet. 2015;386(9994):665-71.

7. Hamza M, Mahmoud N, Elgendy IY. A Randomized Trial of Complete Versus Culprit-Only Revascularization During Primary Percutaneous Coronary Intervention in Diabetic Patients With Acute ST Elevation Myocardial Infarction and Multi Vessel Disease. J Interv Cardiol. 2016;29(3):241-7.

8. Smits PC, Abdel-Wahab M, Neumann FJ, Boxma-de Klerk BM, Lunde K, Schotborgh CE, et al. Fractional Flow Reserve-Guided Multivessel Angioplasty in Myocardial Infarction. N Engl J Med. 2017;376(13):1234-44.

9. Mehta SR, Wood DA, Storey RF, Mehran R, Bainey KR, Nguyen H, et al. Complete Revascularization with Multivessel PCI for Myocardial Infarction. N Engl J Med. 2019.
